# Supplementary material for: Novel genome-wide associations for anhedonia, genetic correlation with psychiatric disorders, and polygenic association with brain structure
Source: Transl Psychiatry. 2019 Dec 4;9:327. doi: 10.1038/s41398-019-0635-y (PMC6892870; doi:10.1038/s41398-019-0635-y)
Supplement: Supplementary file 1 — supplementary legends [file 41398_2019_635_MOESM1_ESM.docx]

Table S1 List of exclusion criteria for the MRI analyses.

**Table S2** Genomic loci associated with anhedonia in the discovery sample. SNP = lead SNP for the Peak, CHR = chromosome number, BP = Base position of lead SNP, A1 = minor frequency allele, A2 = other allele, Beta = coefficient for the lead SNP, S.E. = Standard Error for beta, p = p value, start_BP = start of associated region (r^2^ > 0.1 with lead SNP in 500kb window), stop_bp = end of associated region (r^2^ > 0.1 with lead SNP in 500kb window), info = imputation score, Candidate Genes = genes that may be the cause of the association.

**Figure S1** Plot of independent locus associating with anhedonia. X axis = position of chromosome, Y axis = -log10(p), purple region = associated region (r^2^ > 0.1 with lead SNP in 500kb window) with lead SNP.

**Figure S2** Plot of independent locus associating with anhedonia. X axis = position of chromosome, Y axis = -log10(p), purple region = associated region (r^2^ > 0.1 with lead SNP in 500kb window) with lead SNP.

**Figure S3** Plot of independent locus associating with anhedonia. X axis = position of chromosome, Y axis = -log10(p), purple region = associated region (r^2^ > 0.1 with lead SNP in 500kb window) with lead SNP.

**Figure S4** Plot of independent locus associating with anhedonia. X axis = position of chromosome, Y axis = -log10(p), purple region = associated region (r^2^ > 0.1 with lead SNP in 500kb window) with lead SNP.

**Figure S5** Plot of independent locus associating with anhedonia. X axis = position of chromosome, Y axis = -log10(p), purple region = associated region (r^2^ > 0.1 with lead SNP in 500kb window) with lead SNP.

**Figure S6** Plot of independent locus associating with anhedonia. X axis = position of chromosome, Y axis = -log10(p), purple region = associated region (r^2^ > 0.1 with lead SNP in 500kb window) with lead SNP.

**Figure S7** Plot of independent locus associating with anhedonia. X axis = position of chromosome, Y axis = -log10(p), purple region = associated region (r^2^ > 0.1 with lead SNP in 500kb window) with lead SNP.

**Figure S8** Plot of independent locus associating with anhedonia. X axis = position of chromosome, Y axis = -log10(p), purple region = associated region (r^2^ > 0.1 with lead SNP in 500kb window) with lead SNP.

**Figure S9** Plot of independent locus associating with anhedonia. X axis = position of chromosome, Y axis = -log10(p), purple region = associated region (r^2^ > 0.1 with lead SNP in 500kb window) with lead SNP.

**Figure S10** Plot of independent locus associating with anhedonia. X axis = position of chromosome, Y axis = -log10(p), purple region = associated region (r^2^ > 0.1 with lead SNP in 500kb window) with lead SNP.

**Figure S11** Plot of independent locus associating with anhedonia. X axis = position of chromosome, Y axis = -log10(p), purple region = associated region (r^2^ > 0.1 with lead SNP in 500kb window) with lead SNP.

**Table S3** Genomic loci associated with anhedonia in the replication sample. SNP = lead SNP for the Peak, CHR = chromosome number, BP = Base position of lead SNP, A1 = minor frequency allele, A2 = other allele, Beta = coefficient for the lead SNP, S.E. = Standard Error for beta, p = p value.

Table S4 Linear regression models adjusted for age, age^2^, sex, three scanner brain position variables (lateral, transverse and longitudinal position), genotype array and the first eight genetic principal components. CSF = cerebrospinal fluid.

Table S5 Coefficients are from linear mixed models adjusted for age, age^2^, sex, total brain volume, three scanner brain position variables (lateral, transverse and longitudinal position), genotype array, hemisphere and the first eight genetic principal components. ROI volumes are grey matter volumes (mm3), apart from subcortical regions (amygdala, caudate, nucleus accumbens, putamen, hippocampus) where volumes are for the whole structure. Pseudo r^2^ is Efron's r^2^.

Figure S12 ROIs showing association with anhedonia PRS (see Table S3). Probabilistic Harvard-Oxford cortical/subcortical ROIs (thresholded at 25%) obtained via FMRIB Software Library are overlaid onto the MNI-152 template in MRIcroGL (<http://www.mccauslandcenter.sc.edu/mricrogl/>). All regions showed negative association with the anhedonia PRS.

**Table S6** Linear regression models adjusted for age, age^2^, sex, three scanner brain position variables (lateral, transverse and longitudinal position), genotype array and the first eight genetic principal components. FA = fractional anisotropy; MD = mean diffusivity.

**Table S7** Coefficients are for linear mixed models adjusted for age, age^2^, sex, three scanner brain position variables (lateral, transverse and longitudinal position), genotype array, hemisphere and the first eight genetic principal components. *For non-bilateral tracts, coefficients are for linear regression models adjusted for all above covariates apart from hemisphere. Pseudo r^2^ is Efron's r^2^.

**Table S8** Coefficients are for linear mixed models adjusted for age, age^2^, sex, three scanner brain position variables (lateral, transverse and longitudinal position), genotype array, hemisphere and the first eight genetic principal components. *For non-bilateral tracts, coefficients are for linear regression models adjusted for all above covariates apart from hemisphere. Pseudo r^2^ is Efron's r^2^.

**Table S9** Linear regression models adjusted for age, age^2^, sex, head motion during fMRI, three scanner brain position variables (lateral, transverse and longitudinal position), genotype array and the first eight genetic principal components. Group-defined mask includes occipito-temporal and amygdalar regions.
