# Supplementary material for: Novel genome-wide associations for anhedonia, genetic correlation with psychiatric disorders, and polygenic association with brain structure
Source: Transl Psychiatry. 2019 Dec 4;9:327. doi: 10.1038/s41398-019-0635-y (PMC6892870; doi:10.1038/s41398-019-0635-y)
Supplement: Supplementary file 2 — supplementary methods [file 41398_2019_635_MOESM2_ESM.docx]

### Supplementary methods

### MRI acquisition and pre-processing, and IDP selection

#### Tissue and ROI volumes

Structural and functional brain MRI was collected at a single site (Manchester) using a Siemens Skyra 3T scanner with a 32-channel head coil. A 3D MPRAGE sequence was used to acquire T1-weighted structural images at 1 mm^3^ resolution. Grey matter, white matter and CSF volumes, as well as volumes of 139 ROIs, were derived using FMRIB’s Automated Segmentation Tool^1^.

Associations were examined between anhedonia PRS and grey matter volumes of 10 cortical ROIs, and total volumes of five subcortical ROIs. As a meta-analysis of brain structure and anhedonia is not yet available, 12 of the ROIs were selected based on regions reported as showing reliable evidence of reduced volume in major depressive disorder (MDD) vs. healthy controls in a recent voxel-based meta-analysis^2^. Three further ROIs (nucleus accumbens, putamen, amygdala) were selected based on previous studies reporting association between measures of anhedonia and structural volumes^3-7^. UK Biobank ROIs are defined according to Harvard-Oxford cortical and subcortical atlases. The atlas region corresponding to the peak MNI coordinates reported in previous articles was employed.

#### DTI measures of white matter tract integrity

A Stejskal-Tanner pulse sequence (fov = 104x104x72; TE = 92 ms) was used to acquire diffusion weighted images. Gradient distortion correction was applied, and data were corrected for eddy currents and head motion using FSL’s Eddy tool (<http://fsl.fmrib.ox.ac.uk/fsl/fslwiki/EDDY>). Modelling of within-voxel tract structure using BEDPOSTx and PROBTRACKx enabled derivation of DTI measures of weighted-mean tract FA (the fraction of water molecule diffusion that is directionally coherent in a tract; larger values reflect better white matter integrity) and MD (the mean extent of diffusion in three axes; larger values reflect poorer integrity) for 27 white matter tracts.

General components of the weighted-mean FA and MD (*g*FA; *g*MD) values were derived using principal component analysis across the 27 white matter tract IDPs^33,34^. Scores were extracted for the first unrotated component: this first component (consisting of a linear combination of all tracts) accounted for 42.16% of the total variance in FA (n = 14,481), and 46.62% in MD (n = 14,191).

#### Task fMRI

fMRI data were acquired using a gradient-echo echo planar imaging (GE-EPI) sequence (fov = 88x88x64; TE = 39 ms; TR = 0.735 s). Participants completed a 4 minute version of the Hariri face/shape emotion processing task^8,9^, where they are presented with pairs of faces (with angry or fearful expressions) or shapes at the bottom of the screen, and are asked to select which image matches the face/shape presented at the top of the screen. Trials were presented in blocks of six trials of the same image type (face or shape).

Pre-processing applied to data collected during this task involved motion correction; spatial smoothing; intensity normalisation; high-pass temporal filtering; EPI unwarping; and gradient distortion correction unwarping. Modelling of task-related activation was conducted using FEAT (FMRI Expert Analysis Tool). Of the activation contrasts applied, we were most interested in the Face – Shape contrast (subtracting activity during shape processing from that during angry/fearful face processing), measuring emotional reactivity^9^. In generating IDPs, two ROIs were defined, the first based on voxels showing group-level fixed-effect z-statistics of > 120 for the faces-shapes contrast, conducted in the first 5000 MRI participants. This encompassed primarily amygdala and occipito-temporal regions. In the second mask, the same thresholding was applied, but within an amygdala mask. Here we focussed on two UK Biobank IDPs based on median blood oxygen level dependent (BOLD) activity across voxels within each of these ROIs.

For further details on MRI variables see https://biobank.ctsu.ox.ac.uk/crystal/docs/brain_mri.pdf

Associations of the PRS (standardised) with each MRI outcome were assessed in linear regression models, or, in the case of bilateral individual tracts/ROIs, linear mixed models. MRI outcomes were standardised, and so results reflect differences measured in standard deviation units. All cortical/subcortical ROIs, and most individual white matter tracts (all except forceps major, forceps minor and middle cerebellar peduncle) were bilateral, and so linear mixed models first tested for interactions between PRS and hemisphere – none were significant, and so results presented for these outcomes include hemisphere as a fixed factor without the hemisphere*PRS interaction.

1. Zhang Y, Brady M, Smith S. Segmentation of brain MR images through a hidden Markov random field model and the expectation-maximization algorithm. *IEEE Transactions on Medical Imaging.* 2001;20(1):45-57.

2. Wise T, Radua J, Via E, et al. Common and distinct patterns of grey-matter volume alteration in major depression and bipolar disorder: evidence from voxel-based meta-analysis. *Mol Psychiatry.* 2017;22(10):1455-1463.

3. Pizzagalli DA, Holmes AJ, Dillon DG, et al. Reduced caudate and nucleus accumbens response to rewards in unmedicated individuals with major depressive disorder. *Am J Psychiatry.* 2009;166(6):702-710.

4. Wacker J, Dillon DG, Pizzagalli DA. The role of the nucleus accumbens and rostral anterior cingulate cortex in anhedonia: integration of resting EEG, fMRI, and volumetric techniques. *Neuroimage.* 2009;46(1):327-337.

5. Auerbach RP, Pisoni A, Bondy E, et al. Neuroanatomical Prediction of Anhedonia in Adolescents. *Neuropsychopharmacology : official publication of the American College of Neuropsychopharmacology.* 2017;42(10):2087-2095.

6. Harvey PO, Pruessner J, Czechowska Y, Lepage M. Individual differences in trait anhedonia: a structural and functional magnetic resonance imaging study in non-clinical subjects. *Mol Psychiatry.* 2007;12(8):703, 767-775.

7. Günther V, Lindner C, Dannlowski U, Kugel H, Suslow T. Amygdalar Gray Matter Volume and Social Relating in Schizophrenia. *Neuropsychobiology.* 2016;74(3):139-143.

8. Hariri AR, Tessitore A, Mattay VS, Fera F, Weinberger DR. The amygdala response to emotional stimuli: a comparison of faces and scenes. *Neuroimage.* 2002;17(1):317-323.

9. Barch DM, Burgess GC, Harms MP, et al. Function in the human connectome: task-fMRI and individual differences in behavior. *Neuroimage.* 2013;80:169-189.

10. Sergerie K, Chochol C, Armony JL. The role of the amygdala in emotional processing: a quantitative meta-analysis of functional neuroimaging studies. *Neurosci Biobehav Rev.* 2008;32(4):811-830.
