# Supplementary figures and images for: Novel genome-wide associations for anhedonia, genetic correlation with psychiatric disorders, and polygenic association with brain structure
Source: Transl Psychiatry. 2019 Dec 4;9:327. doi: 10.1038/s41398-019-0635-y (PMC6892870; doi:10.1038/s41398-019-0635-y)

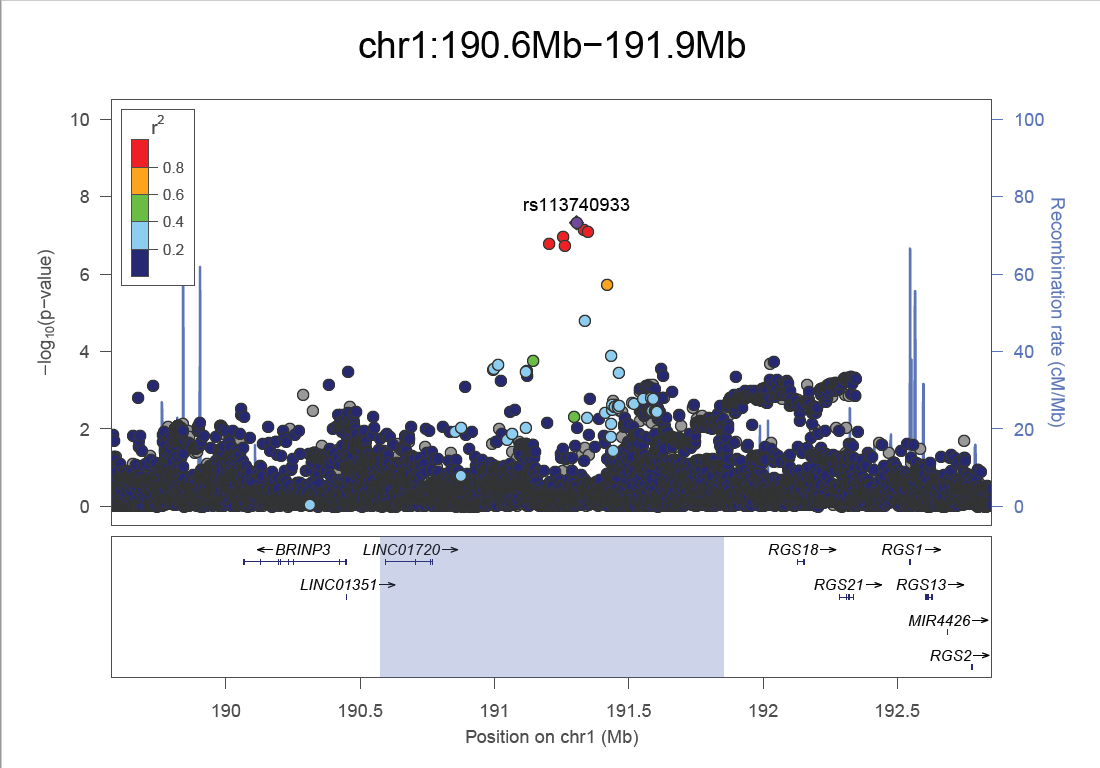

Supplement: Supplementary file 5 — Figure S1 [file 41398_2019_635_MOESM5_ESM.tif]

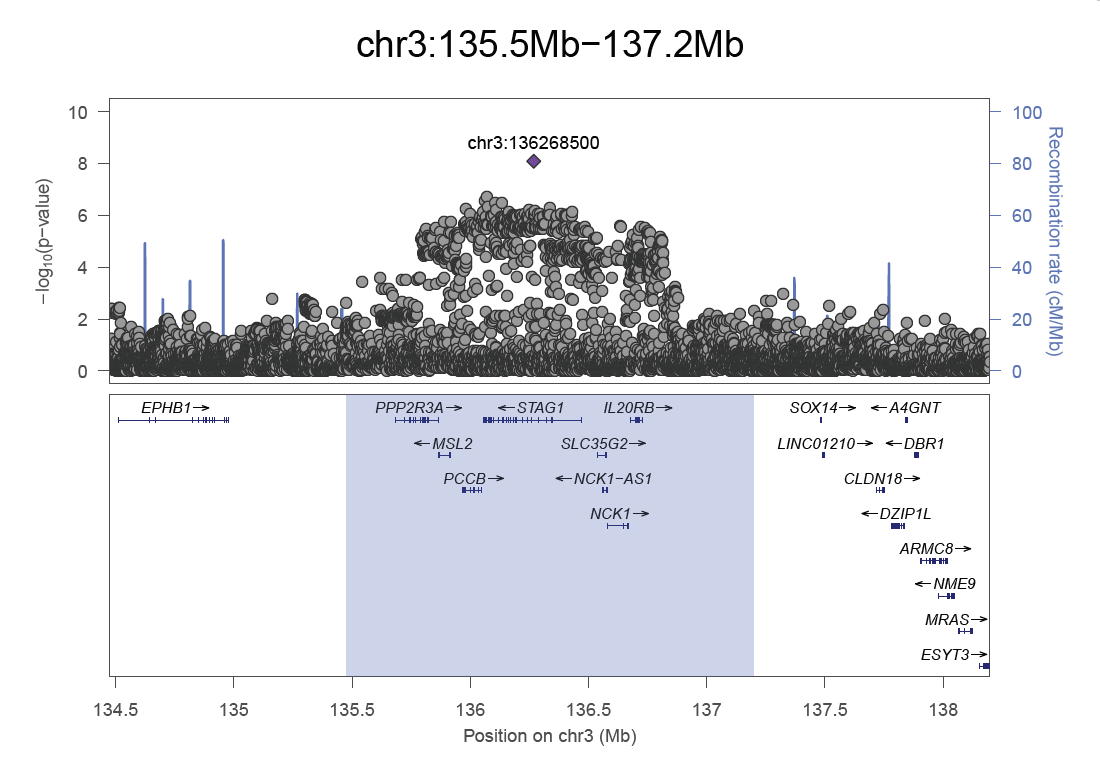

Supplement: Supplementary file 6 — Figure S2 [file 41398_2019_635_MOESM6_ESM.tif]

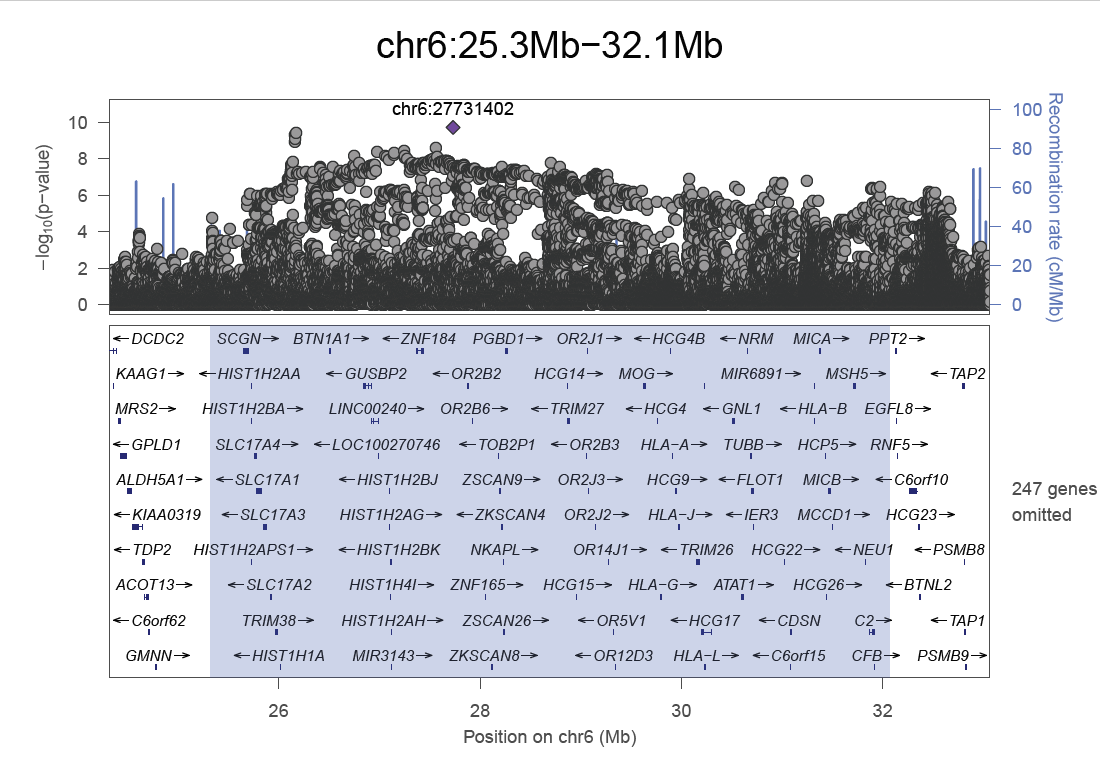

Supplement: Supplementary file 7 — Figure S3 [file 41398_2019_635_MOESM7_ESM.tif]

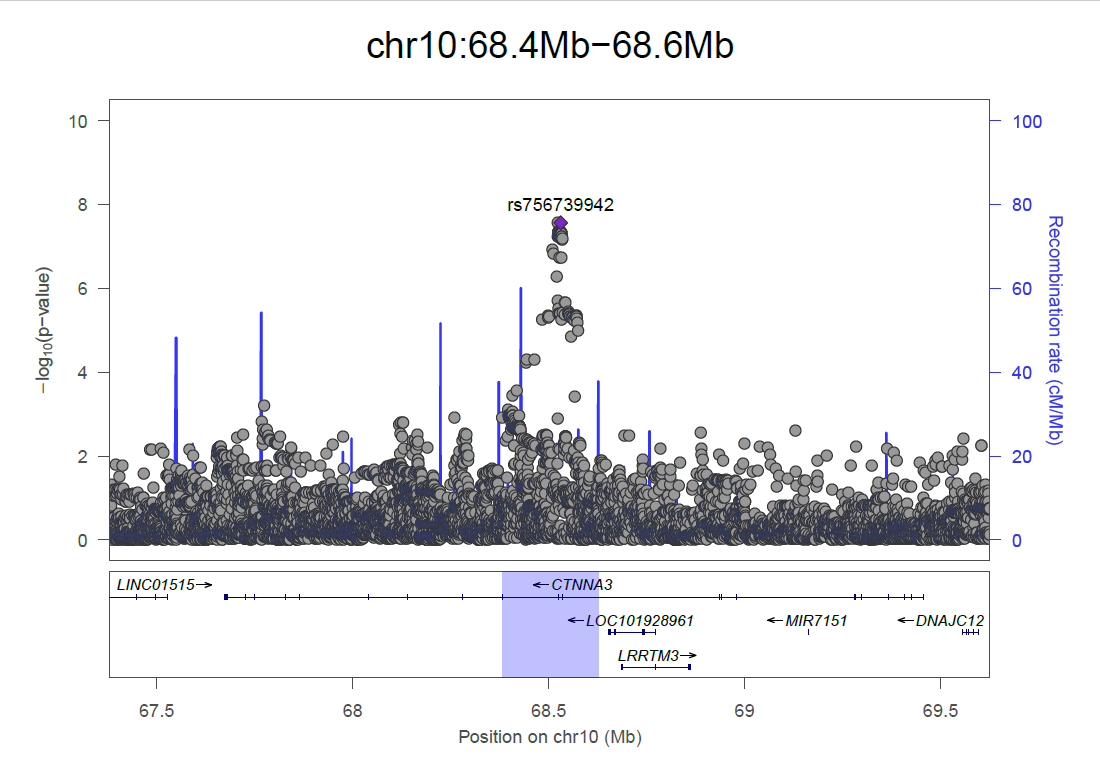

Supplement: Supplementary file 8 — Figure S4 [file 41398_2019_635_MOESM8_ESM.tif]

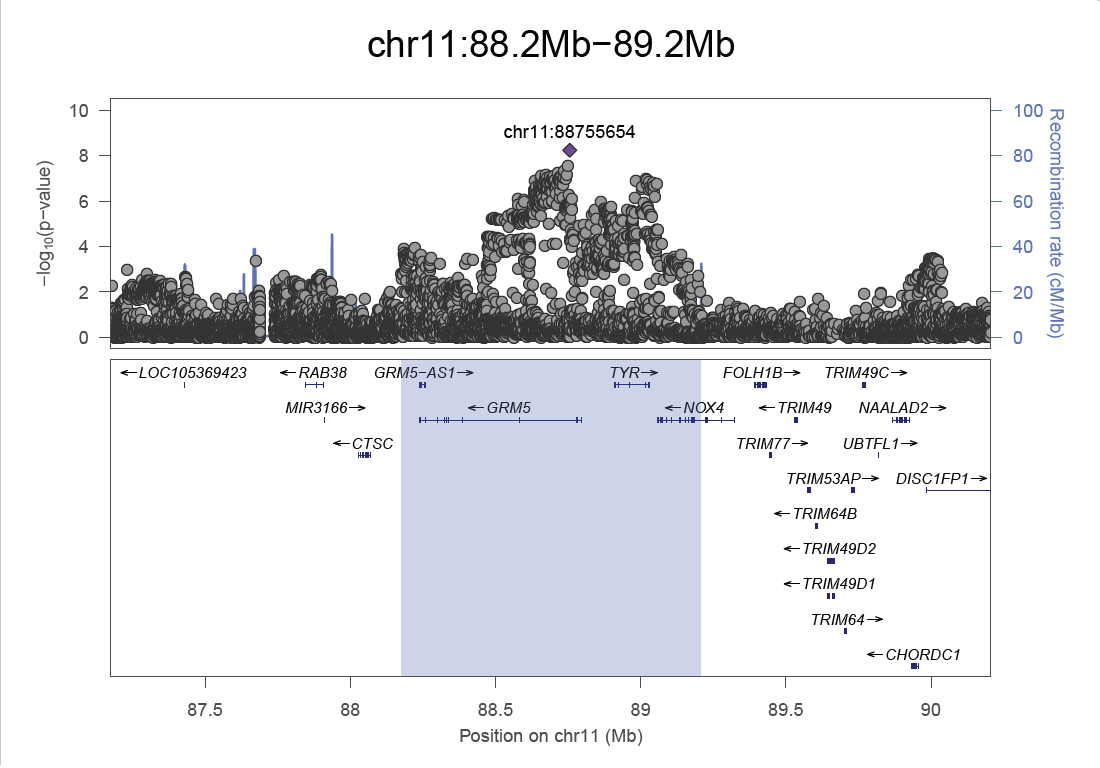

Supplement: Supplementary file 9 — Figure S5 [file 41398_2019_635_MOESM9_ESM.tif]

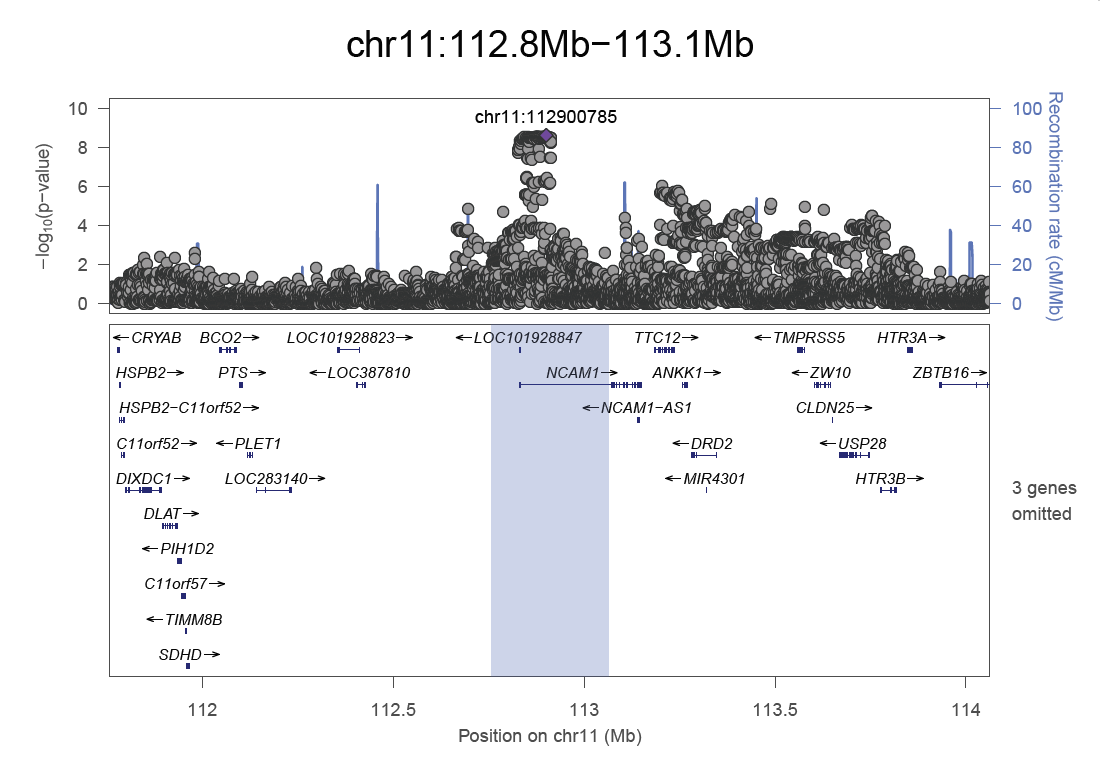

Supplement: Supplementary file 10 — Figure S6 [file 41398_2019_635_MOESM10_ESM.tif]

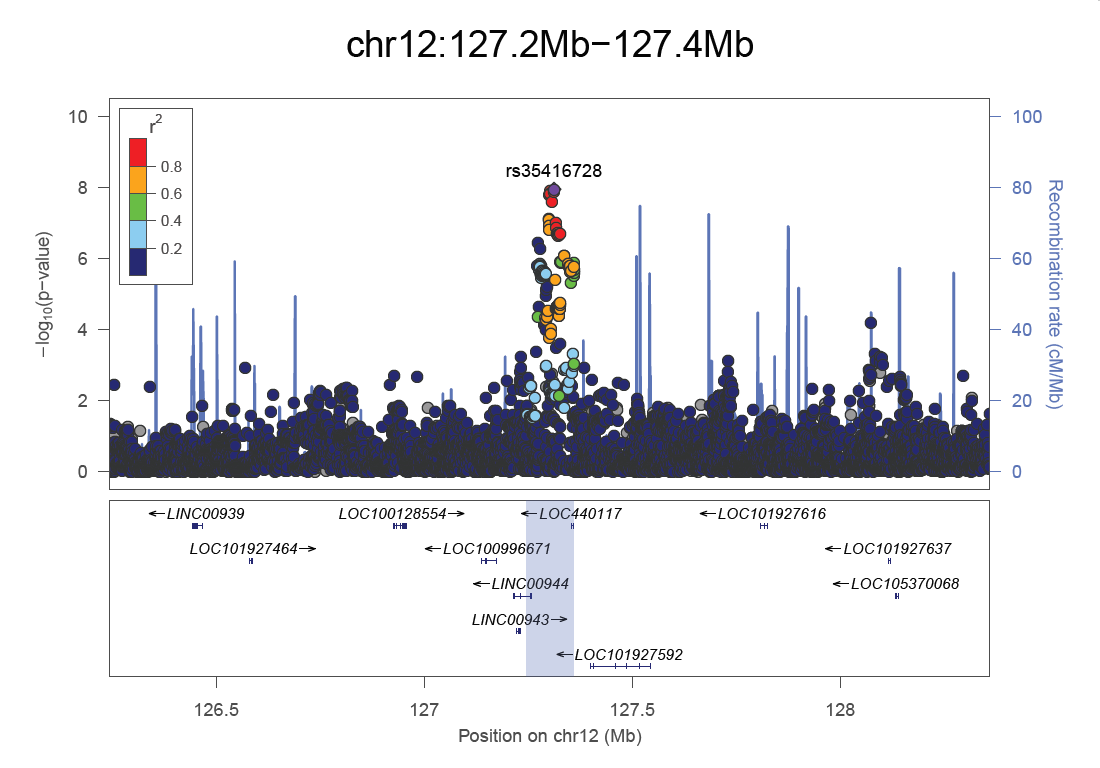

Supplement: Supplementary file 11 — Figure S7 [file 41398_2019_635_MOESM11_ESM.tif]

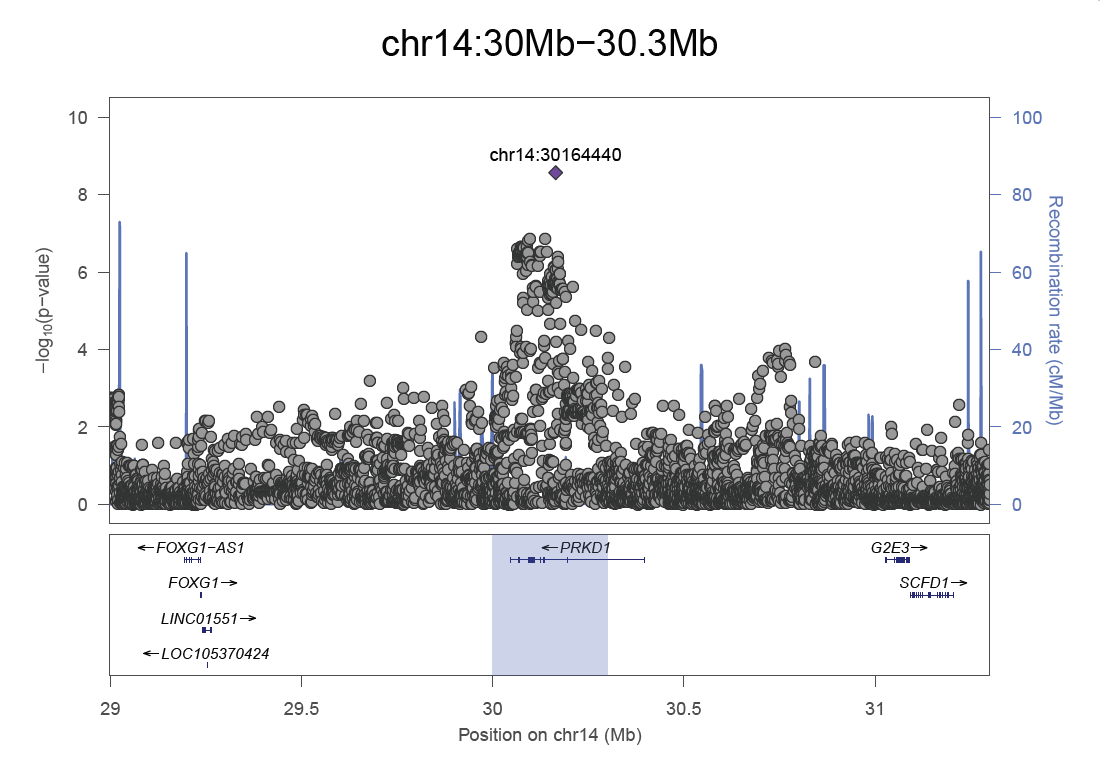

Supplement: Supplementary file 12 — Figure S8 [file 41398_2019_635_MOESM12_ESM.tif]

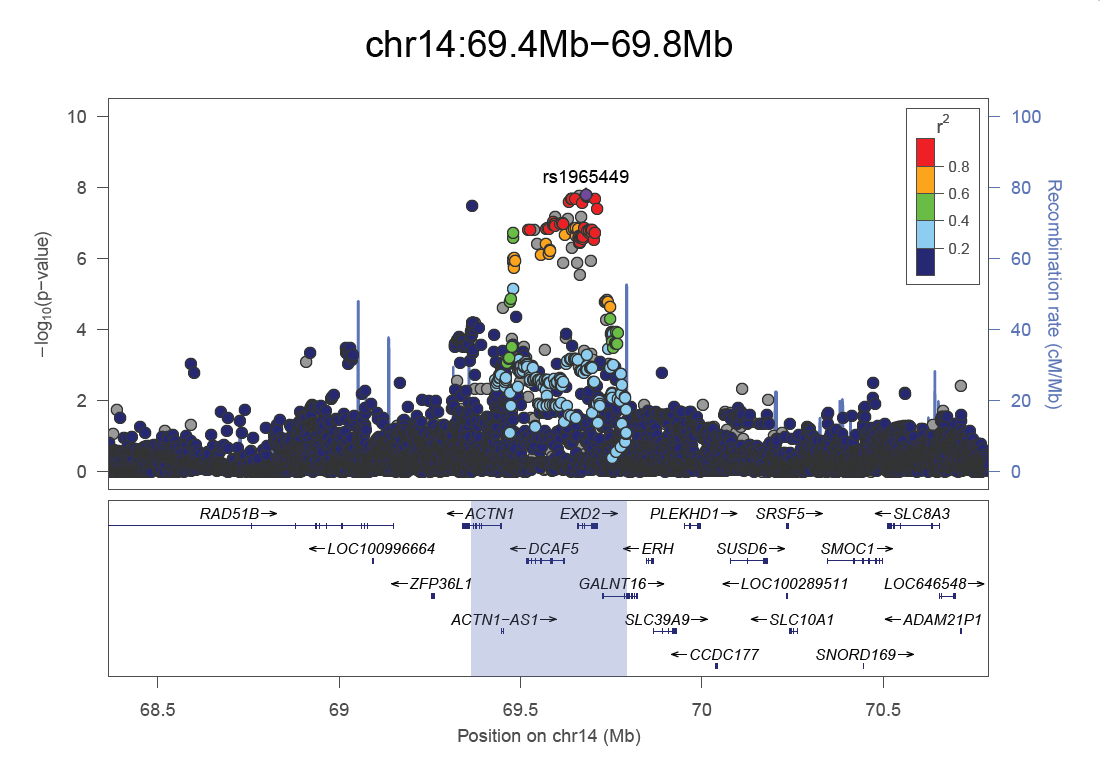

Supplement: Supplementary file 13 — Figure S9 [file 41398_2019_635_MOESM13_ESM.tif]

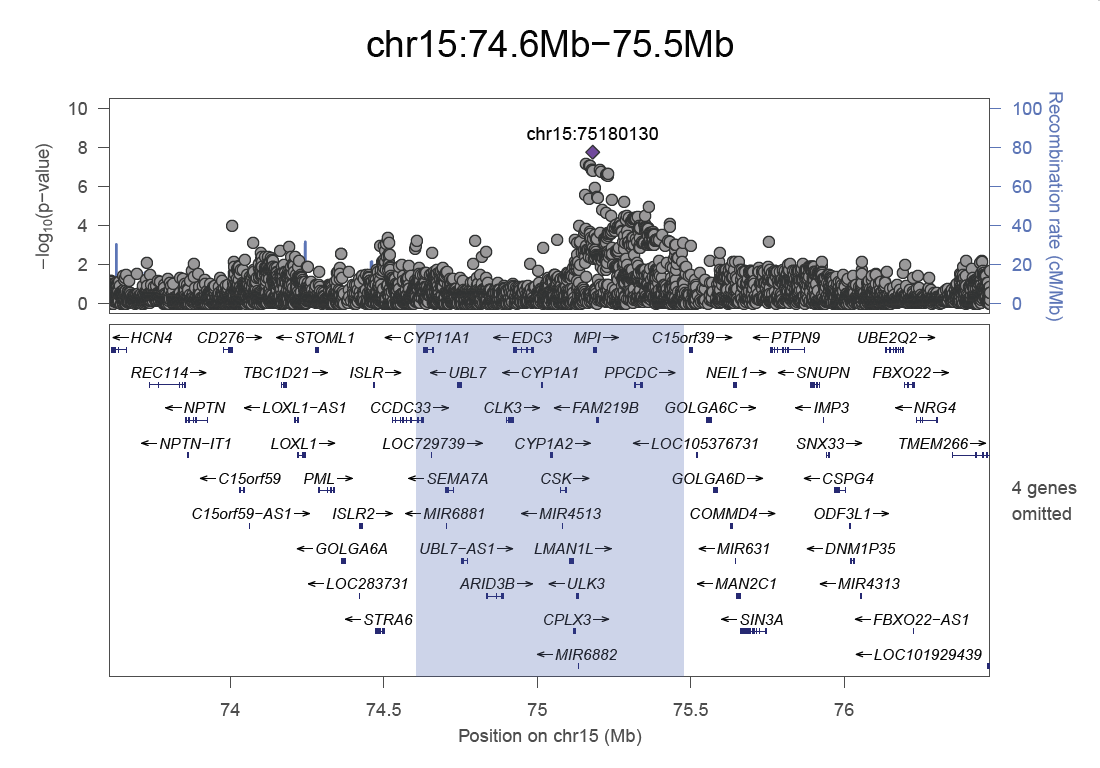

Supplement: Supplementary file 14 — Figure S10 [file 41398_2019_635_MOESM14_ESM.tif]

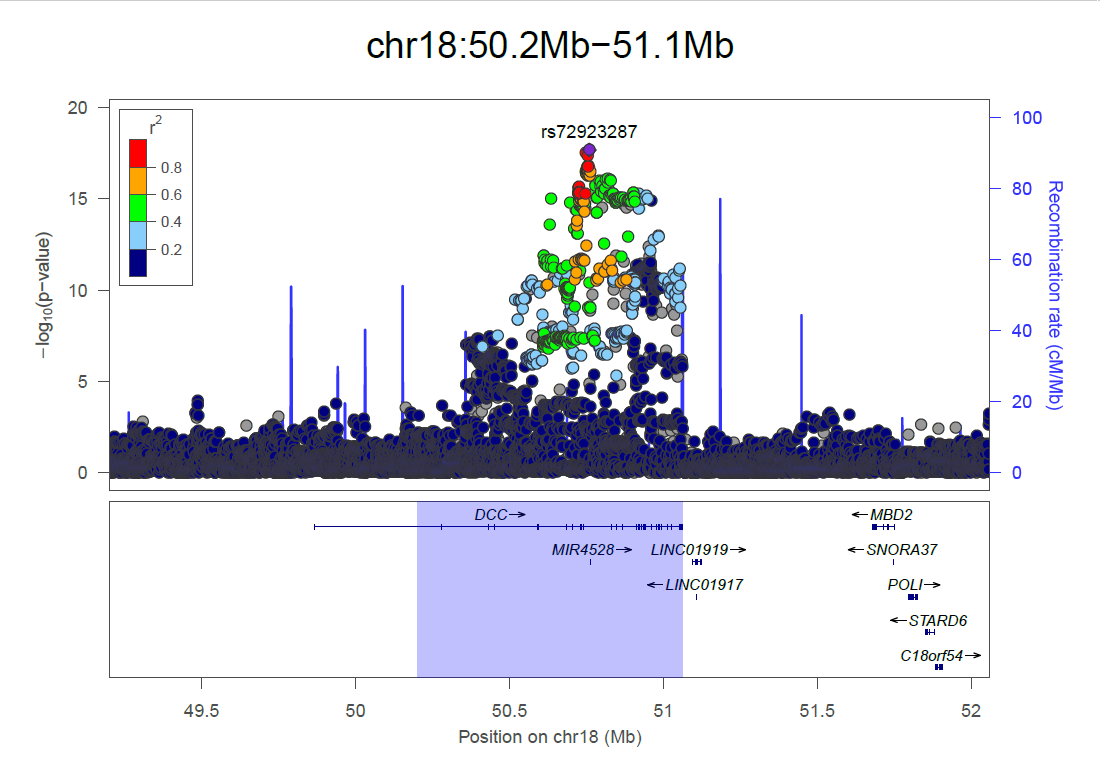

Supplement: Supplementary file 15 — Figure S11 [file 41398_2019_635_MOESM15_ESM.tif]

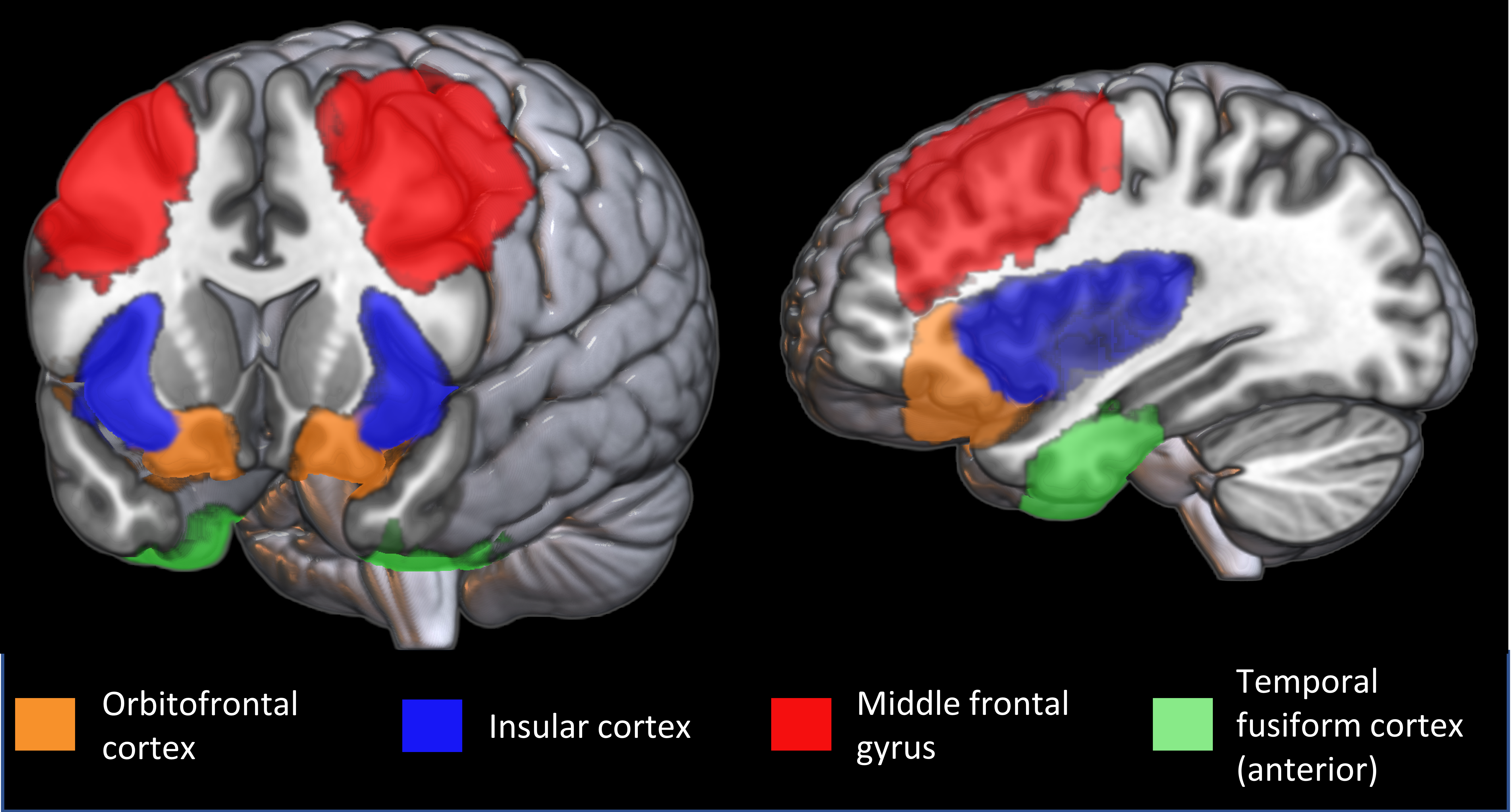

Supplement: Supplementary file 19 — Figure S12 [file 41398_2019_635_MOESM19_ESM.tif]
